# Mesenteric SMARCA2-Deficient Yet SMARCA4-Preserved Aggressive Undifferentiated Tumor: A Case Report

**DOI:** 10.70352/scrj.cr.24-0070

**Published:** 2025-02-20

**Authors:** Ichiro Tamaki, Koichi Kitagawa, Hidetaka Kozai, Yoshikuni Yonenaga, Takashi Nitta

**Affiliations:** 1 Department of Surgery, Ako City Hospital, Ako, Hyogo, Japan; 2 Department of Oncology and Hematology, Hyogo Prefectural Harima-Himeji General Medical Center, Himeji, Hyogo, Japan

**Keywords:** rhabdoid tumor, mesenteric undifferentiated tumor, SMARCA2, SMARCA4, SWItch Sucrose non-fermentable chromatin remodeling complex

## Abstract

**INTRODUCTION:**

The SWItch Sucrose Non-Fermentable (SWI/SNF) chromatin remodeling complex, which includes components such as SMARCA4 and SMARCA2, regulates gene expression by controlling chromatin compaction and accessibility in an ATP-dependent manner. These components are also implicated in carcinogenesis. Thoracic SMARCA4-deficient undifferentiated tumor is a recently introduced category in the fifth edition of the WHO classification in 2021, typically exhibiting rhabdoid morphology in adults. In contrast, rhabdoid tumors occurring within the abdominal cavity in adults are rare and sporadic, with limited detailed documentation, making them relatively less understood compared to their thoracic counterparts.

**CASE PRESENTATION:**

A man in his 70s was admitted to our hospital with a chief complaint of fever. He was diagnosed with a mesenteric solid tumor measuring 6 cm in maximum diameter. Shortly after the hospitalization, bowel obstruction became evident, accompanied by the rapid tumor progression, and then surgical treatment was attempted. A soft, bulky tumor situated in the mesentery accompanied by extensive tumor dissemination was found intraoperatively. The tumor was resected along with the obstructed terminal ileum, aiming to restore intestinal patency and obtain tissue samples. Histopathologically, the tumor represented morphological features resembling a rhabdoid tumor along with a high Ki67 labeling index (50%). Immunohistochemistry revealed SMARCA2 deficiency with preserved SMARCA4 expression. The absence of Claudin-4 expression further supported the diagnosis of a mesenteric SMARCA2-deficient yet SMARCA4-preserved undifferentiated tumor. The patient succumbed 20 days after surgery due to aggressive peritonitis carcinomatosis.

**CONCLUSIONS:**

To the best of our knowledge, this is the first case report of a mesenteric undifferentiated tumor with rhabdoid cytomorphology due to SWI/SNF chromatin remodeling complex deficiency caused by isolated SMARCA2 deficiency. The tumor, in our case, arose in the abdominal organs and appears to share a similar oncogenic process with the category of thoracic SMARCA4-deficient undifferentiated tumors in the WHO classification. Further research is required to improve our understanding of its clinical features, underlying mechanisms, and optimal management strategies.

## Abbreviations

SWI/SNF
: SWItch Sucrose Non-Fermentable
CECT
: contrast-enhanced CT
NSCLC
: non-small cell lung cancer
TMB
: tumor mutation burden

## INTRODUCTION

Rhabdoid tumors are rare, aggressive malignancies, well recognized as one of the phenotypes of rhabdoid tumor predisposition syndrome in infants and young children, associated with a poor prognosis.^1)^ Le Loarer *et al*. reported 19 cases of thoracic undifferentiated tumors exhibiting rhabdoid morphology caused by SMARCA4 deficiency in adults.^2)^ Thoracic SMARCA4-deficient undifferentiated tumor is a recent category introduced in the fifth edition of the WHO classification in 2021.^3)^ However, reports of rhabdoid tumors within the abdominal cavity are sporadic, with limited detailed documentation, rendering them relatively less understood.

We present a case of a mesenteric undifferentiated tumor characterized by vigorous progression and rhabdoid morphological features histologically. Ultimately, the diagnosis was determined to be due to SWItch Sucrose Non-Fermentable (SWI/SNF) chromatin remodeling complex deficiency resulting from solitary SMARCA2 deficiency.

## CASE PRESENTATION

A man in his 70s was admitted to our hospital due to persistent fever (body temperature: 38.5°C, persisting for 2 weeks) and diarrhea (1 to 2 times a day, persisting for 4 weeks). His appetite and oral food intake were normal during this period. A solid mass, the size of a fist, was palpable on his lower abdomen without associated tenderness. He had a history of heavy smoking (Brinkman index: 800) and was diagnosed with chronic obstructive pulmonary disease.

**Fig. 1:**
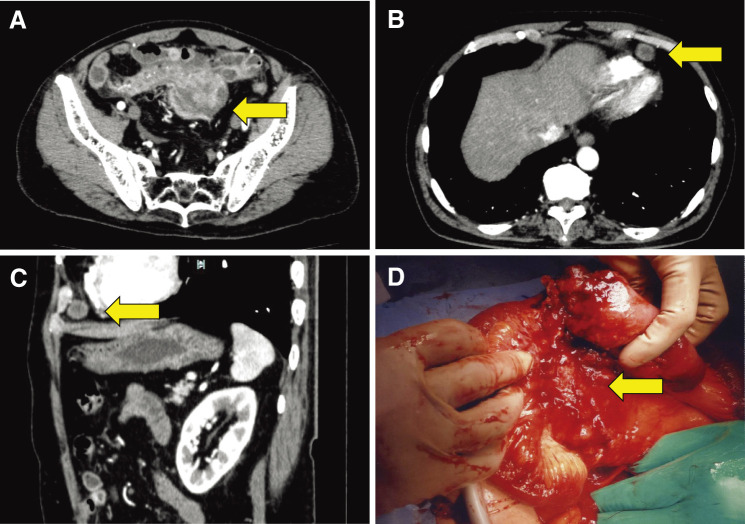
Contrast-enhanced CT (CECT) images upon admission (Day 1) and intraoperative photograph. CECT revealed a solid tumor measuring 6 cm in maximum diameter with moderate vascularity located in the mesentery of the terminal ileum (**A**, arrow) and a mediastinal tumor measuring 1.5 cm in diameter (**B** and **C**, arrow). A soft, bulky tumor situated in the mesentery involved the terminal ileum (**D**, arrow).

Upon admission, contrast-enhanced CT (CECT) revealed a solid tumor measuring 6 cm in maximum diameter with moderate vascularity located in the mesentery of the terminal ileum accompanied by minimal ascites (**Fig. 1A**). Findings indicative of intestinal obstruction were not identified. A mediastinal tumor measuring 1.5 cm in diameter was also detected (**Figs. 1B** and **1C**). Blood tests showed an elevated white blood cell count (15900/mm^3^) and C-reactive protein (32.4 mg/dL). Tumor markers, including carcinoembryonic antigen, CA19-9, and CA15-3, were within normal range, except for CA125 (201.8 U/mL).

Our initial diagnosis was malignant lymphoma involving the digestive tract and mediastinum. Accordingly, the treatment approach was planned to involve histological confirmation and initiation of chemotherapy. However, on the third day following admission, abdominal distension and vomiting became evident, leading to the diagnosis of small bowel obstruction. Gastrointestinal decompression using a long tube was performed; however, it proved to be ineffective. A small bowel contrast study and following abdominal CT demonstrated severe intestinal obstruction caused by the solid tumor. Therefore, surgical treatment was attempted (Day 6).

### Intraoperative findings

A soft, bulky tumor situated in the mesentery and involving the terminal ileum was observed (**Fig. 1D**), accompanied by extensive tumor dissemination towards the sigmoid colon and bladder. Additionally, a moderate volume of ascites with rich bloody components was present across the abdominal cavity. Due to extensive peritoneal tumor dissemination, a palliative surgery was performed to obtain a tissue diagnosis and relieve intestinal obstruction, as an R0 resection was deemed unfeasible.

### Postoperative clinical course

**Fig. 2:**
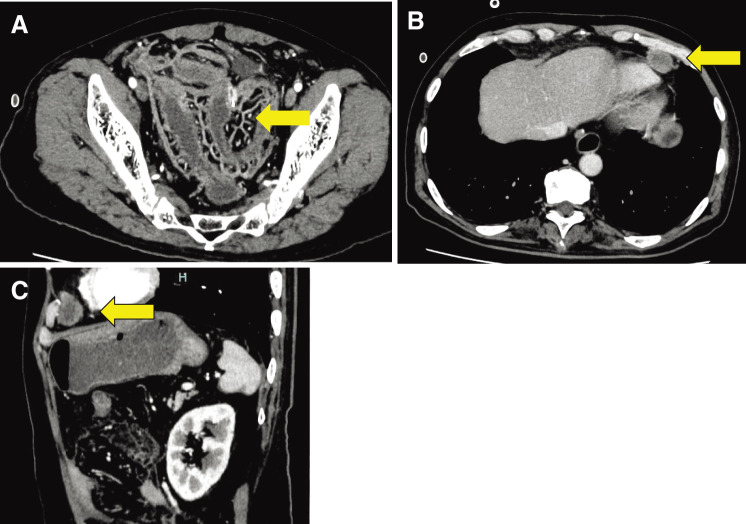
Contrast-enhanced CT (CECT) images (Day 15). The CT study revealed extensive enlargement of pan-mesenteric lymphatic vessels, suggesting rapid lymphovascular invasion by malignant tissue (**A**, arrow). The mediastinal tumor represented a 200% increase in diameter over 15 days (**B** and **C**, arrow).

Despite transient improvement in oral food intake following surgery, the postoperative course was marred by rapid progression of residual tumor tissue. Within a short timeframe, the patient developed symptoms, including fever, diarrhea, and abdominal distention, which progressively worsened over time. The CT study revealed extensive enlargement of pan-mesenteric lymphatic vessels, suggesting rapid lymphovascular invasion by malignant tissue (**Fig. 2A**). Peritonitis carcinomatoses showed day-by-day progression, accompanied by a 200% increase in the diameter of the mediastinal tumor over 15 days (**Figs. 2B** and **2C**). The patient succumbed 20 days after the surgery (Day 26), and a final pathological diagnosis was obtained posthumously.

### Pathological findings

**Fig. 3:**
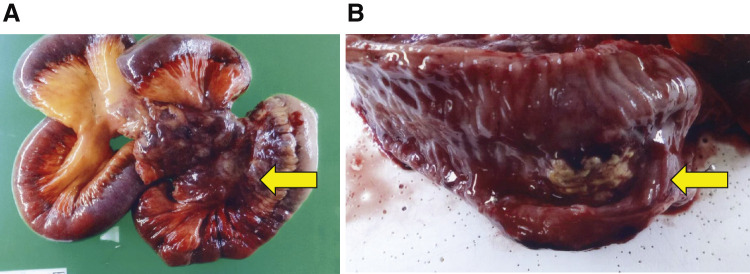
Macroscopic findings of the resected specimen. A fragile, milky-white tumor was present within the mesentery of the small intestine (**A**, arrow), partially penetrating the intestinal wall (**B**, arrow).

**Fig. 4:**
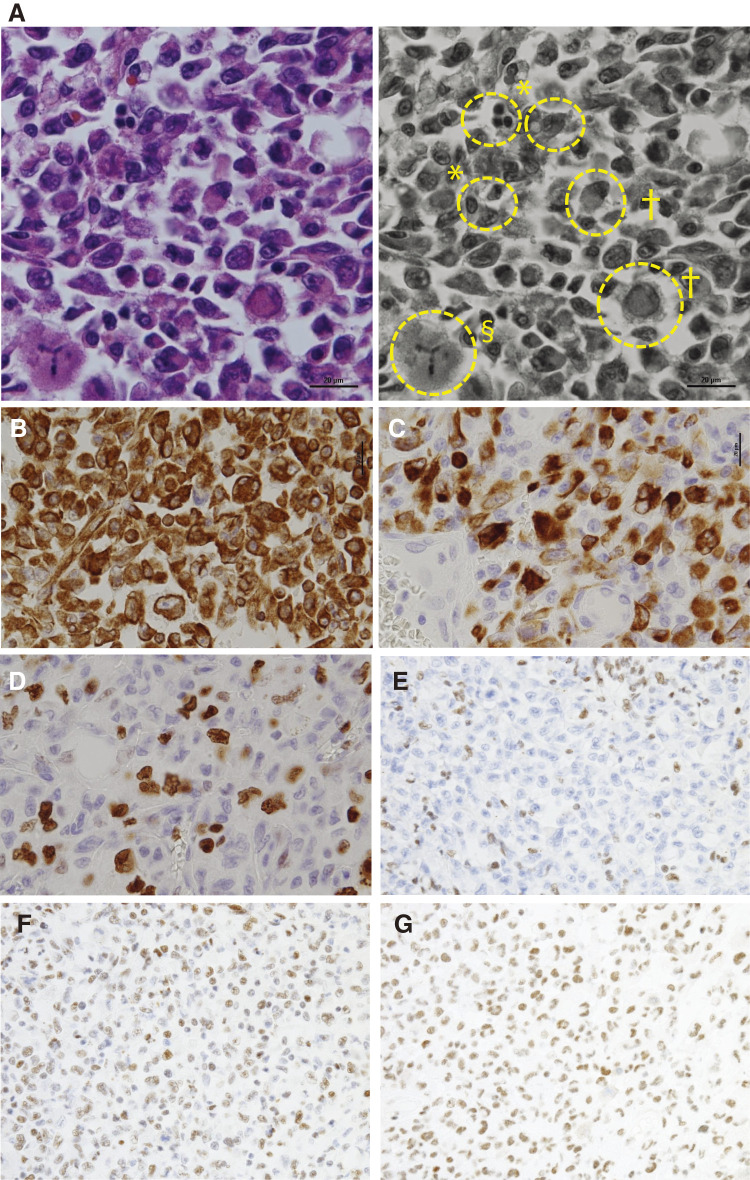
Histopathological findings of the mesenteric tumor. Hematoxylin–eosin staining exhibited diffuse and dense proliferation of round cells with dense chromatin (**A**, *). Wide eosinophilic cells with eccentric nuclei (**A**, †), indicative of morphological features resembling a rhabdoid tumor, and tripolar mitosis (**A**, §) were observed. Immunostaining was diffuse positive for Vimentin (**B**) and focally positive for CK7 (**C**). Ki 67 labeling index was approximately 50% in most parts of the specimen (**D**). Immunohistochemical studies to investigate the SWI/SNF chromatin remodeling complex exhibited deficiency in SMARCA2 (BRM) (**E**) while maintaining proficiency in SMARCA4 (BRG1) (**F**) and SMARCB1(INII) (**G**).

A fragile, milky-white tumor was present within the mesentery of the small intestine, partially penetrating the intestinal wall macroscopically (**Figs. 3A** and **3B**). The histopathological evaluation exhibited an undifferentiated tumor, which was characterized by diffuse and dense proliferation of round, polygonal cells with eccentric nuclei, often displaying dense chromatin, and wide eosinophilic cytoplasm, indicative of morphological features resembling a rhabdoid tumor (**Fig. 4A**). The results of the immunostaining were as follows:

Diffuse positive: Vimentin (**Fig. 4B**)
Focally positive: AE1/AE3, CK7 (**Fig. 4C**), CAM 5.2.
Negative: HMB45, Melan A, CD34, c-kit, CD117, αSMA, S100, desmin, D2-40, calretinin, LCA, CD3, CD20, 34βE12, claudin-4.

Based on these results, the pathologists excluded malignant melanoma, gastrointestinal stromal tumor, malignant lymphoma, and malignant mesothelioma, cancer metastasis, including prostate or pancreas, from the differential diagnosis. Ki 67 labeling index was approximately 50% in most parts of the specimen (**Fig. 4D**). In response to the presence of rhabdoid cells in the tumor, further immunohistochemical studies were conducted to investigate the SWI/SNF chromatin remodeling complex, exhibiting deficiency in SMARCA2 (BRM) while maintaining proficiency in SMARCA4 (BRG1) and SMARCB1(INII) (**Figs. 4E**–**4G**). The absence of claudin-4 expression excluded mesenteric metastasis from non-small cell lung cancer (NSCLC), ultimately resulting in the diagnosis of a SMARCA2-deficient undifferentiated tumor arising in the intestinal mesentery.

## DISCUSSION

In the fifth edition of the WHO classification (2021), a new category of thoracic tumors, thoracic SMARCA4-deficient undifferentiated tumors, was introduced. This tumor is characterized by its high malignancy in adults with primary involvement in the thoracic region and undifferentiated rhabdoid morphology.^3)^

SMARCA4 is one of the 15 components comprising the SWI/SNF chromatin remodeling complex, known to regulate gene expression by controlling chromatin compaction and accessibility for gene transcription in an ATP-dependent manner. In addition to SMARCA4, the SWI/SNF complex also includes SMARCA2 and SMARCB1, each known as constituent components implicated in carcinogenesis.^4)^ Most SWI/SNF-deficient undifferentiated tumors exhibit monomorphic, undifferentiated epithelioid cells with rhabdoid cytomorphology.^4)^

While rhabdoid tumors have been well recognized as highly malignant neoplasms in pediatric patients, with atypical teratoid/rhabdoid tumors arising in the central nervous system,^5)^ recently, tumors displaying characteristic rhabdoid morphology are reported in various organs, including thoracic tumors, among adult malignancies.^6,7)^ Moreover, while SWI/SNF-deficient undifferentiated tumors are rare entities and typically display characteristic rhabdoid morphology, SWI/SNF complex deficiency is observed in over 20% of epithelial malignant tumors in both children and adults, even in the absence of this morphology.^4)^ Associations have been suggested, including differentiation status, MSI mutations, and BRAF mutations.^8)^

Thoracic tumors with SMARCA4 deficiency comprise two subtypes: SMARCA4-deficient undifferentiated tumors and NSCLC with SMARCA4 deficiency.^9)^ Considering the fact that SWI/SNF complex deficiency is found in gastrointestinal malignancies, similar observations may be suggested in other types of cancers. According to Rekhtman *et al*., the epithelial adhesion molecule Claudin-4 is expressed in SWI/SNF-deficient NSCLC, while almost no expression is observed in SWI/SNF-deficient undifferentiated tumors.^10)^ Thus, Claudin-4 may serve as a criterion for determining the origin of tumors.^9)^

Here, we present a case of a patient diagnosed with a mesenteric undifferentiated tumor exhibiting rhabdoid features. The clinical course was notable for the unusually rapid progression of the tumor, distinguishing it from commonly encountered malignant neoplasms. The mesenteric tumor displayed morphological features resembling rhabdoid tumors and exhibited vigorous cell proliferation, prompting consideration of SWI/SNF-deficient tumors in the differential diagnosis. Ultimately, SMARCA2 deficiency was noted, while SMARCA4 expression was preserved. Furthermore, the absence of Claudin-4 expression led to the diagnosis of a Mesenteric SMARCA2-deficient yet SMARCA4-preserved undifferentiated tumor.

While SMARCA4-deficient undifferentiated tumors generally coincide with SMARCA2 deficiency, reports of undifferentiated tumors with preserved SMARCA4 expression and isolated SMARCA2 deficiency are limited. In 2022, Iwakoshi et al. reported three cases of thoracic SMARCA2-deficient yet SMARCA4-preserved undifferentiated tumors exhibiting Claudin-4 deficiency. Similar to our case, all three cases had unfavorable prognoses compared to NSCLC.^9)^

It is reported that SWI/SNF deficiency, including isolated SMARCA2 loss, is present in a certain proportion of gastrointestinal cancers associated with poor prognosis.^11)^ However, reports of gastrointestinal undifferentiated tumors exhibiting rhabdoid features, solitary SMARCA2-deficiency, and Claudin-4 negativity are rare, and to our knowledge, this is the first report of such a case.

Regarding treatment strategies, the therapeutic approach for Thoracic SMARCA4-UT may serve as a useful reference. Cytotoxic chemotherapy is not considered effective in SMARCA4-deficient tumors. Interestingly, SMARCA4 expression is a predictive biomarker in patients undergoing adjuvant platinum-based chemotherapy in NSCLC. Next-generation sequencing of SMARCA4-UT has shown a high tumor mutation burden (TMB) in some cases, suggesting that regimens containing immune checkpoint inhibitors may offer promising treatment strategies.^10,12)^ While publication bias should be taken into account, a durable response to pembrolizumab in thoracic SMARCA4-UT has been reported in a single case.^13)^

Limitations are as follows: Regarding the mediastinal tumor, its consistent enlargement with the mesenteric tumor suggests a correlation; however, a biopsy was not performed. As for the possibility of the mediastinal tumor being a primary tumor metastasizing to the abdominal cavity, this scenario appears less likely given the lack of enlargement of mediastinal lymph nodes or lung tumors, along with the larger tumor volume in the abdominal cavity. Due to the rapid progression of the disease, the diagnosis of the SNF/SWI-deficient mesenteric tumor was made posthumously, preventing the assessment of microsatellite instability status, programmed cell death protein 1/programmed cell ligand 1 expression, and TMB in our case.

## CONCLUSION

In conclusion, evaluation of SWI/SNF expression and Claudin-4 could lead to the final diagnosis of intra-abdominal undifferentiated tumor presenting vigorous growth and rhabdoid morphology. Its clinical presentation is challenging, and the establishment of the concept and nosology of this rare malignancy is recent; further research is required to improve our understanding of its clinical features, underlying mechanisms, and optimal management strategies. When encountering an abdominal undifferentiated tumor with aggressive growth, SWI/SNF-deficient UT should be considered as a potential diagnosis, as this entity remains relatively novel and not well recognized outside of thoracic organs.

## ACKNOWLEDGMENTS

We would like to express our sincere gratitude to Dr. Tokiko NAKAI and Dr. Tomonori OTANI for their invaluable effort in the pathological examination. Their dedication and expertise greatly contributed to the final diagnosis of the case.

## DECLARATIONS

### Funding

Not applicable.

### Authors’ contributions

IT contributed to the study conception, data collection, and writing.

KK, HK, YY, and TN contributed to critical review and revision.

All authors gave final approval of the article and accepted accountability for all aspects of the work.

### Availability of data and materials

Not applicable.

### Ethics approval and consent to participate

Not applicable.

### Consent for publication

Informed consent was obtained from the patient’s family to publish this case report.

### Competing interests

The authors declare that they have no competing interests.
